# Comparison of CrN Coatings Prepared Using High-Power Impulse Magnetron Sputtering and Direct Current Magnetron Sputtering

**DOI:** 10.3390/ma16186303

**Published:** 2023-09-20

**Authors:** Heda Bai, Jin Li, Jialai Gao, Jinyang Ni, Yaxiong Bai, Jie Jian, Lin Zhao, Bowen Bai, Zeyun Cai, Jianchao He, Hongsheng Chen, Xuesong Leng, Xiangli Liu

**Affiliations:** 1 School of Materials Science and Engineering, Harbin Institute of Technology (Shenzhen), Shenzhen 518055, China; 20b955008@stu.hit.edu.cn (H.B.); 22B955003@stu.hit.edu.cn (J.N.); 2 Institute of Special Environments Physical Sciences, Harbin Institute of Technology (Shenzhen), Shenzhen 518055, China; 21S155061@stu.hit.edu.cn (J.G.); 22S155088@stu.hit.edu.cn (Y.B.); jianjie@hit.edu.cn (J.J.); zhaolin2020@hit.edu.cn (L.Z.); baibowen@hit.edu.cn (B.B.); caizeyun@hit.edu.cn (Z.C.); hejianchao@hit.edu.cn (J.H.); chenhongsheng@hit.edu.cn (H.C.)

**Keywords:** CrN, coatings, high power impulse magnetron sputtering, direct current magnetron sputtering, microstructure, mechanical, irradiation behaviors

## Abstract

Chromium Nitride (CrN) coatings have widespread utilization across numerous industrial applications, primarily attributed to their excellent properties. Among the different methods for CrN coating synthesis, direct current magnetron sputtering (DCMS) has been the dominant technique applied. Nonetheless, with the expanded applications of CrN coatings, the need for enhanced mechanical performance is concurrently escalating. High-power impulse magnetron sputtering (HiPIMS), an innovative coating deposition approach developed over the past three decades, is gaining recognition for its capability of yielding coatings with superior mechanical attributes, thereby drawing significant research interest. Considering that the mechanical performance of a coating is fundamentally governed by its microstructural properties, a comprehensive review of CrN coatings fabricated through both techniques is presented. This review of recent literature aims to embark on an insightful comparison between DCMS and HiPIMS, followed by an examination of the microstructure of CrN coatings fabricated via both techniques. Furthermore, the exploration of the underlying factors contributing to the disparities in mechanical properties observed in CrN coatings is revealed. An assessment of the advantages and potential shortcomings of HiPIMS is discussed, offering insight into CrN coating fabrication.

## 1. Introduction

**Figure 1:**
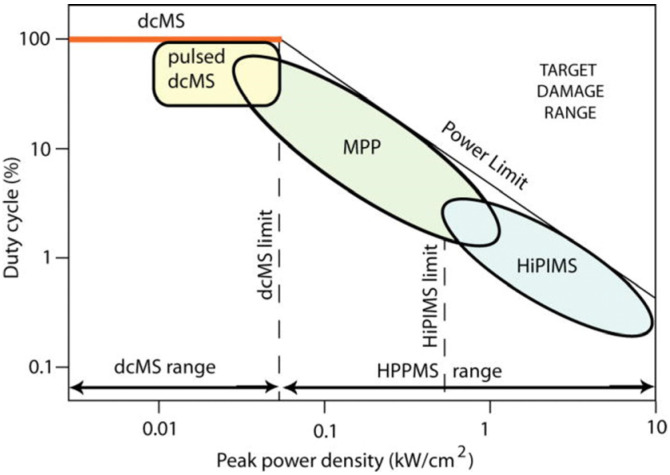
A schematic comparison of duty cycle and peak power density on magnetron sputtering [34]. HiPIMS demonstrates a higher peak power density compared to DCMS. Other approaches include modulating the pulse such that in the initial stages of the pulse (a few hundred microseconds), the power level is moderate (typical for a DCMS), followed by a high power pulse (lasting a few hundred microseconds up to a millisecond), which is referred to as modulated pulse power (MPP); the duty cycle and peak power density of MMP are between DCMS and HiPIMS [34]. Reprinted with permission from [34] the American Vacuum Society 2012.

**Figure 2:**
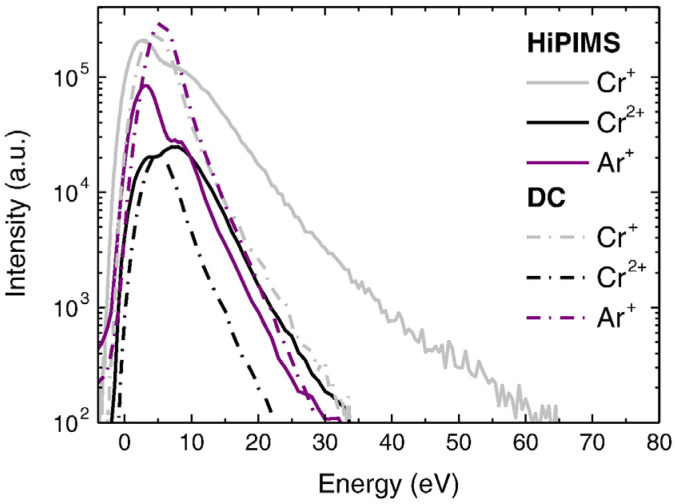
Time-integrated ion energy distributions of ionic species detected using the HiPIMS and DCMS discharges [35]. The concentration of high-energy target ions in HiPIMS surpasses that in DCMS, while the working gas ion concentration and energy distribution between the two methods are comparable. Reprinted with permission from [35] Elsevier 2014.

Transition metal nitrides constitute a category of interstitial compounds formed through the insertion of nitrogen atoms at interstitial positions within the crystal lattice. These compounds simultaneously exhibit properties characteristic of covalent compounds and ionic crystals. The introduction of nitrogen atoms results in alterations of chemical bonding energies, lattice expansion, increased interatomic distances, and a larger lattice constant. These modifications confer unique physical and chemical properties upon such compounds, thereby qualifying transition metal nitrides for distinctive scientific consideration [1,2,3]. Chromium Nitride (CrN) coating is renowned for its exceptional properties and has found extensive utility across numerous industrial applications. One standout characteristic of CrN coating is its exceptional hardness [4,5,6,7,8], rendering it highly resistant to wear and abrasion, and hence advantageous in scenarios where the coated surface encounters high frictional forces [9,10,11], such as for cutting tools, molds, and forming dies. Moreover, CrN coating also exhibits high adhesion to a variety of substrate materials [12,13,14,15], including stainless steel, titanium (Ti), aluminum (Al), etc. The high adhesion strength ensures that the coating remains intact even under severe mechanical stresses, thus offering enduring protection to the underlying substrate. Furthermore, the CrN coating demonstrates a low coefficient of friction [16,17,18]. This particular trait is beneficial in applications demanding minimal friction and enhanced lubricity, such as sliding or rotating components. In addition to these properties, the coating possesses commendable chemical stability, corrosion resistance, and oxidation resistance [19,20,21,22,23,24,25]. This is achieved by forming a protective barrier that prevents the underlying material from reacting with the corrosive substances. Hence, CrN coating is particularly suitable for applications in corrosive environments or ones frequently exposed to moisture and chemicals. In summation, CrN coating integrates the virtues of hardness, adhesion, low friction, corrosion resistance, and thermal stability, making it a versatile coating for an array of industrial applications. The choice of fabrication techniques can markedly affect the microstructure and mechanical properties of the coating. CrN is typically deposited using physical vapor deposition (PVD) techniques. Magnetron sputtering is the most frequently employed technique. The outcome is a high-density coating with excellent adhesion and uniform thickness. This technique allows precise control over coating properties, such as composition and microstructure [17,19,26,27,28,29]. Direct current magnetron sputtering (DCMS) is a conventional sputtering technique that has seen widespread application over the years. In DCMS, a direct current power supply is used to generate a plasma discharge within a vacuum chamber, created by applying a DC voltage between the target material (the material to be sputtered) and the substrate. It typically operates at relatively low power densities and is commonly used for the deposition of metallic and compound coatings. DCMS presents several advantages, including simplicity, cost-effectiveness, and ease of operation. It ensures good coating adhesion and thickness control, making it versatile for various industrial applications. Nevertheless, DCMS suffers from shortcomings such as low ionization efficiency and inadequate control over the energy and momentum of the ions, leading to limited control over coating properties such as density, microstructure, and residual stress. In contrast, high-power impulse magnetron sputtering (HiPIMS) is an advanced novel sputtering technique that has been gaining traction in recent years [30,31,32,33]. It is essentially an upgrade of conventional DCMS. HiPIMS employs short and high-intensity power pulses directed at the target material [34]. These pulses typically last for microseconds and boast high peak power levels (Figure 1). HiPIMS enables the regulation of peak power by manipulating pulse duration and frequency. The plasma density increases with increased power density supplied to the target. Therefore, the coating performance can be improved by the selection of HiPIMS parameters. HiPIMS produces smooth coatings characterized by enhanced friction resistance. The high power density of the cathode yields high-energy particles (Figure 2), which result in hard, high-density coatings exhibiting impressive wear resistance [35]. In addition, HiPIMS offers advantages such as improved coating density, reduced droplet formation, and enhanced adhesion over DCMS. HiPIMS also enables better control over ion energy, utilizing the accelerating effect of the bias voltage field on ions, which facilitates tailoring coating properties and the deposition of complex material (such as nitrides and oxides using reactive sputtering or alloys grown with multi-target sputtering) [36,37,38,39].

In this review, the microstructural characteristics of CrN coatings prepared via DCMS and HiPIMS are discussed. The mechanical performance disparities resulting from these microstructural attributes are analyzed. Relevant literature from recent years is reviewed, aiming to guide researchers regarding the exploitation of CrN coatings, along with HiPIMS and DCMS techniques. The merits and potential limitations of HiPIMS are appraised, providing insights into the fabrication of CrN coatings.

## 2. Plasma-Based Depositions

### 2.1. Plasma Characteristics

The ionization efficiency during the PVD sputtering deposition processes significantly influences the formation of coatings profoundly impacts coating formation. The ionization efficiency refers to the proportion of sputtered atoms ionized in the plasma, influencing the coating’s adhesion, microstructure, and attributes. A higher ionization efficiency generally yields enhanced coating properties [40,41]. Furthermore, heightened ionization rates can alter the coating’s microstructure. Due to the higher kinetic energy of the ions, their impact on the surface of the coating effectively increases the energy of the adatoms. This amplified energy increases adatoms’ mobility significantly, fostering enhanced surface diffusion and the reorganization of adatoms during coating growth. Consequently, it contributes to augmented coating density, a smoother surface morphology, and defect reduction [36,37,42,43]. A higher ionization efficiency can yield coatings with superior mechanical and tribological properties. Hence, controlling and optimizing the ionization efficiency during PVD sputtering is vital for achieving the desired coating formation and performance [36,44,45,46,47].

**Figure 3:**
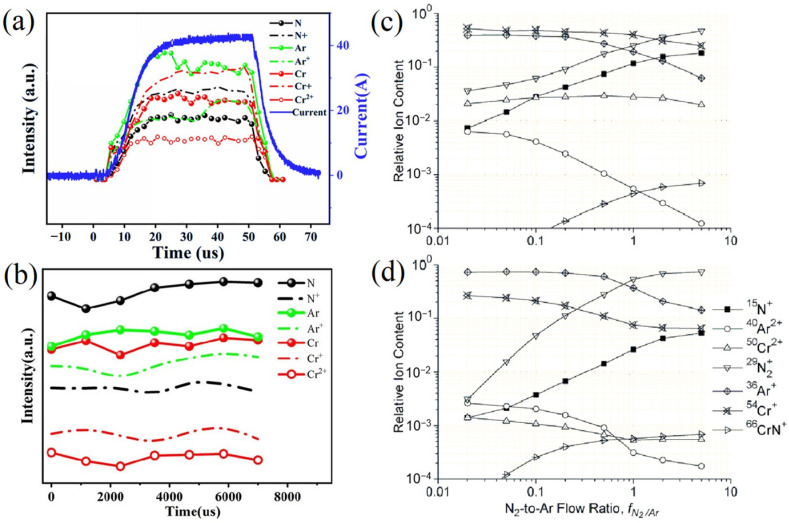
The time-resolved OES of selected radicals in (**a**) HiPIMS (50 μs, 200 Hz) and (**b**) DCMS [48]; Relative ion content in the flux incident upon the substrate during (**c**) HiPIMS and (**d**) DCMS process, plotted versus the N_2_-to-Ar flow ratio f_N2/Ar_ [49]. Compared to DCMS, the HiPIMS plasma contains a higher abundance of Cr ions, even surpassing the concentration of the working gas ions. Reprinted with permission from [48] AIP Publishing 2020, [49] IEEE Xplore 2010.

Li et al. employed time-resolved optical emission spectroscopy (OES) to diagnose the species in DCMS and HiPIMS plasma near the surface of targets [48]. Figure 3a reveals the plasma variation with the current in both HiPIMS and DCMS discharge. The primary species in HiPIMS plasma were Cr^+^ ions, while neutral Cr and N dominated the DCMS plasma. The calculated ratios of (Cr^+^ + Cr^2+^)/Cr and N^+^/N in HiPIMS were 1.85 and 1.41, respectively, compared to 0.49 and 0.48 in DCMS. This discrepancy in ionization rate ratios resulted in HiPIMS plasma having an overall higher energy than that of DCMS. Greczynski G et al. investigated the relative composition of the ion flux impinging on the growing coating during HiPIMS and DCMS discharge as a function of the N_2_-to-Ar ratio f_N2/Ar_ [49]. As shown in Figure 3c,d, Cr^+^ ions make up the most significant contribution up to f_N2/Ar_ = 2 (where the N^2+^ signal takes over), contrasting the DCMS case, where working gas ions dominate the ion flux to the substrate. This result aligns with Li et al.’s findings [48]. Furthermore, the relative intensity of the Cr^2+^ signal is markedly higher during HiPIMS operation compared to DCMS. Previous studies suggest that higher valence state ions possess greater energy, particularly when biased, which may interrupt the continuous growth of columnar crystals. The continuous growth of columnar crystals can induce shadowing effects, leading to a decrease in the coating density. This often has a negative impact on the mechanical properties of the material. Therefore, interrupting the continuous growth of columnar crystals by bombarding them with high-energy particles is also one of the advantageous characteristics of HiPIMS [50,51,52,53,54].

In conclusion, an increased ion flux leads to more intensive energy bombardment on the substrate. This energy drives coating growth, especially in ceramic coatings like CrN, where atoms must surmount energy barriers for migration and diffusion during the nucleation and growth process. HiPIMS naturally exhibits an inherent advantage in increasing ionization efficiency.

### 2.2. Deposition Rate

Deposition rate, although not an independent process parameter—being a consequence of various factors such as target power, pressure, target-to-substrate distance, and bias voltage—plays a crucial role in the production efficiency and cost-effectiveness of the PVD process. Gaining insights into the deposition rate allows manufacturers to fine-tune process parameters to attain higher deposition rates without jeopardizing the quality of the coating [43,54]. This understanding also aids researchers in unraveling the underpinning mechanisms during deposition, including nucleation, surface diffusion, and grain growth, thereby facilitating the development of models to anticipate coating growth behavior. 

**Figure 4:**
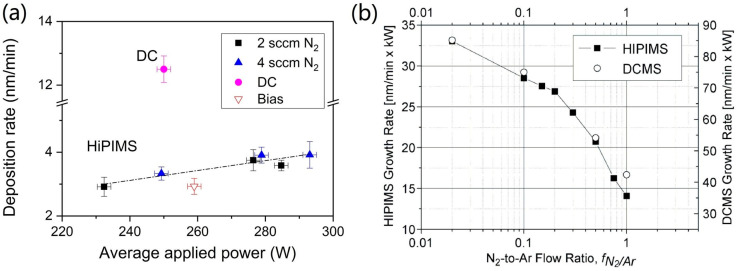
(**a**) The relationship between the deposition rate and the average applied power for the investigated CrN coatings [15]. (**b**) The CrN coatings’ deposition rates of HiPIMS and DCMS at different N_2_-Ar flow ratios (f_N2/Ar_) [49]. At identical average applied power, the deposition rate of CrN coatings obtained via HiPIMS is approximately 1/4 to 1/2 of that achieved by DCMS. Reprinted with permission from [15] Elsevier 2020, [49] IEEE Xplore 2010.

As shown in Figure 4a, J.C. Sánchez-López and colleagues compared the deposition rate of CrN coatings via HiPIMS and DCMS. The research found that the deposition rate of CrN coatings increased linearly with the average target power. However, when a bias was applied, a slight decrease was observed compared to the linear regression of the remaining HiPIMS coatings [15]. Intriguingly, at equivalent average target power, the DC sample exhibited a four-fold augmentation in the deposition rate compared to the HiPIMS sample. It was observed that bias reduced the deposition rate of the CrN coatings prepared via HiPIMS, possibly due to the high plasma ionization rate, which amplified the coating density under the bias effect, consequently decreasing the deposition rate [35], or possibly because of the re-sputtering effect [55]. Greczynski et al. [49] examined the deposition rate of CrN using two techniques, as a function of the N_2_-to-Ar ratio f_N2/Ar_ at the same average target power. As illustrated in Figure 4b, the deposition rate decreased with the increasing of f_N2/Ar_ due to factors like nitride formation on the target surface during the sputtering process (poisoning effect) [56,57] and lower sputtering yield when N_2_ gas partially replaces Ar during the target sputtering process [58,59]. This phenomenon is common in reactive sputtering. Interestingly, the relative drop in deposition rate with an increasing f_N2/Ar_ is analogous in both sputtering techniques, maintaining a consistent multiplier relationship (the deposition rate of DCMS technology being 3–4 times that of HiPIMS technology).

It is recognized that the deposition rate decreases in HiPIMS due to the back-attraction of a large population of positively charged target ions generated during the HiPIMS discharge (the back-sputtering phenomenon refers to the process during target discharge where ions sputtered from the target surface are subsequently drawn back to the target under the influence of a high target voltage, thereby participating in the subsequent sputtering process) [45,60,61,62]. This effect is pronounced for materials with a low sputtering yield [63]. The relatively low deposition rate of HiPIMS also poses a major hindrance to its broad commercial adoption. However, in recent years, advancements have been made in improving the deposition rate of the HiPIMS technique, which will be further elaborated on in Section 5.

## 3. The Influence of HiPIMS and DCMS Techniques on the Growth of CrN Coatings

### 3.1. Texture

Studying the crystallinity and grain size of CrN can be immensely significant for multiple reasons. Primarily, CrN crystallinity directly influences its mechanical, electrical, and optical attributes. A well-established crystalline structure amplifies the material’s hardness and thermal stability, rendering it appropriate for wear-resistant coatings, electronic devices, and optoelectronic applications [13,27,64]. Secondly, the CrN’s grain size directly influences its mechanical properties and surface morphology. Fine-grained CrN coatings display superior hardness, smoothness, and uniformity. Properties are sought for applications demanding low friction, high durability, and premium surface finish [10,28,65]. Additionally, comprehension of the relationship between crystallinity and grain size can provide valuable insights into the growth mechanisms and structural evolution of CrN coatings during the deposition processes [66,67,68,69,70,71]. 

**Figure 5:**
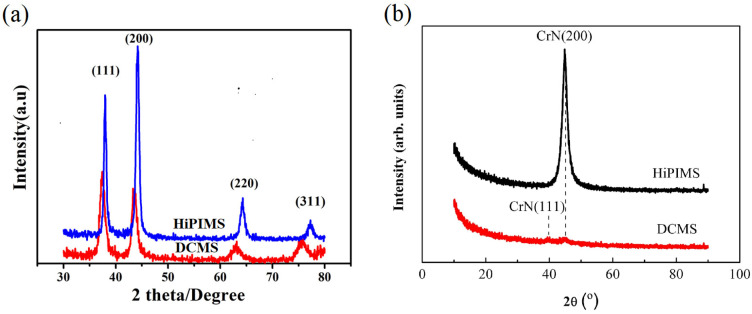
XRD patterns of the CrN by HiPIMS and DCMS at the same average power of (**a**) 0.3 kW [48] and (**b**) 4 kW [72]. The diffraction peak intensity of the CrN coating prepared by HiPIMS surpasses that of DCMS, indicating superior crystallinity. Reprinted with permission from [48] AIP Publishing 2020, [72] Elsevier 2020.

Li et al. [48] studied thr XRD diffraction patterns of CrN coatings deposited with equal average target power via DCMS and HiPIMS. As shown in Figure 5a, the HiPIMS-deposited coating displayed substantially higher intensities of various crystal-oriented diffraction peaks compared to the DCMS-deposited CrN, signifying better crystallinity in the coating. A parallel phenomenon was observed in the study by Zhang et al. [72]. As shown in Figure 5b, HiPIMS-deposited CrN coatings exhibited better crystallinity, while diffraction peaks of DCMS-deposited CrN coatings were considerably weaker, indicating that the DCMS coating has low crystallinity and an incomplete crystal structure. The subsequent high-resolution transmission electron microscopy (HRTEM) confirmed the presence of a significant number of amorphous structures in CrN prepared by DCMS. The hardness tests also showed that CrN deposited by HiPIMS had a higher hardness than that of DCMS [72].

The mechanism underlying the enhancement of mechanical properties of CrN coatings with superior crystallinity resides in the formation of an orderly crystal lattice structure. With higher crystallinity, the crystal grains in CrN coatings are more densely packed and display improved interconnectivity. This results in fortified atomic bonding and increased interatomic forces within the coating. Therefore, a higher degree of crystallinity typically translates to improved mechanical properties such as hardness, wear resistance, and adhesion.

### 3.2. Residual Stress

Thin coating stress, also known as internal stress, denotes the inherent mechanical forces present within a coating material. This stress can arise due to the structural and energetic attributes of the coating material. During coating growth, the deposition of atoms or molecules on the substrate prompts an internal structure rearrangement [73,74,75,76,77,78]. Concurrently, the substrate undergoes bombardment from energetic particles or atoms during deposition, resulting in momentum transfer and stress generation [79,80,81,82,83,84]. 

Coating stress can be broadly categorized into two types: compressive stress and tensile stress. These stresses originate from distinct factors and bear differing effects on the mechanical behavior of coatings. Compressive stress arises from the bombardment of energetic particles (neutrals and/or ions) during film growth, leading to grain boundary densification and dislocation generation. The lattice constant experiences a decrease due to the high density of grain deformation induced by defects and dislocations. Such stress typically bolsters the mechanical performance of coatings, enhancing their hardness and deformation resistance and making them more resistant to wear, fatigue, and scratching. When subjected to external forces or loads, compressive stress can provide support and prevent the coating from plastic deformation [77,78,81,81,84,85,86,87,88]. Therefore, appropriate compressive stress is beneficial for enhancing the mechanical properties of coatings. During the process of thin film deposition, the presence of defects such as vacancies or dislocations can lead to an expansion of the lattice constant, consequently inducing tensile stress within the coating. This can also happen if the coating material has a lower atomic density. The tensile stress in the coating results in the coating being stretched, possibly causing coating rupture, cracking, or the formation of voids [74,75,76,77,88,89].

**Figure 6:**
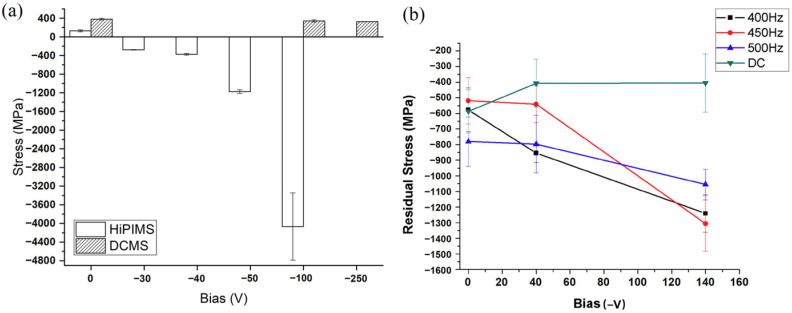
The comparison of the residual stress of CrN coatings prepared by HiPIMS and DCMS as a function of (**a**) different bias voltages [90] and (**b**) HiPIMS frequency [93]. The CrN coating deposited by DCMS exhibits a weak correlation with changes in bias voltage and stress values. Conversely, the stress in the CrN coating deposited by HiPIMS is significantly influenced by substrate bias. Reprinted with permission from [90] Elsevier 2020, [93] Elsevier 2018.

Elo Rg et al. [90] utilized the curvature method to compare the internal stress of CrN coatings deposited via DCMS and HiPIMS techniques under different bias voltages without heating. They employed Stoney’s equation [91,92] for this analysis. Their study indicated that the impact of bias voltage on the internal stress of DCMS-deposited CrN coatings was relatively minor. In contrast, the effect of bias voltage on the internal stress of HiPIMS-deposited CrN coatings was quite substantial (Figure 6a). A study examining the relationship between HiPIMS frequency and the microstructure of CrN thin coatings by Guimaraes et al. identified similar trends [93]. Their findings suggest that increasing the bias values for HiPIMS deposition results in an elevation of compressive stress. The combination of higher peak power from the HiPIMS source and increased bias enhances the ion flux and promotes the compaction of the coatings, leading to elevated residual stresses (Figure 6b).

The CrN coating deposited by DCMS lacks sufficient impact energy on the coating, due to its low ion-to-atom ratio, resulting in a weak correlation with changes in bias voltage and relatively low stress values. The stress level of the coating is predominantly determined by other parameters. Conversely, the stress in the CrN coating deposited by HiPIMS is significantly influenced by substrate bias. This is because a substantial amount of Cr is ionized, deriving energy from the applied substrate bias, leading to extremely high compressive stress generated within the coating [81,87,94].

### 3.3. Micromorphology

Cross-sectional morphology can uncover potential defects in the coating, such as cracks, voids, or inclusions. The inspection of these defects provides valuable information regarding the coating’s quality, mechanical stability, and potential failure mechanisms [6,20,95,96,97,98]. This allows researchers to detect and rectify these defects to enhance the coating’s performance and reliability. In the case of coating failure or performance degradation, cross-sectional images can aid in identifying the root cause. They allow researchers to investigate the failure mode, such as delamination, cracking, or deformation, and probe the factors contributing to the failure. This information is instrumental in devising strategies to improve coating durability and reliability [99]. 

**Figure 7:**
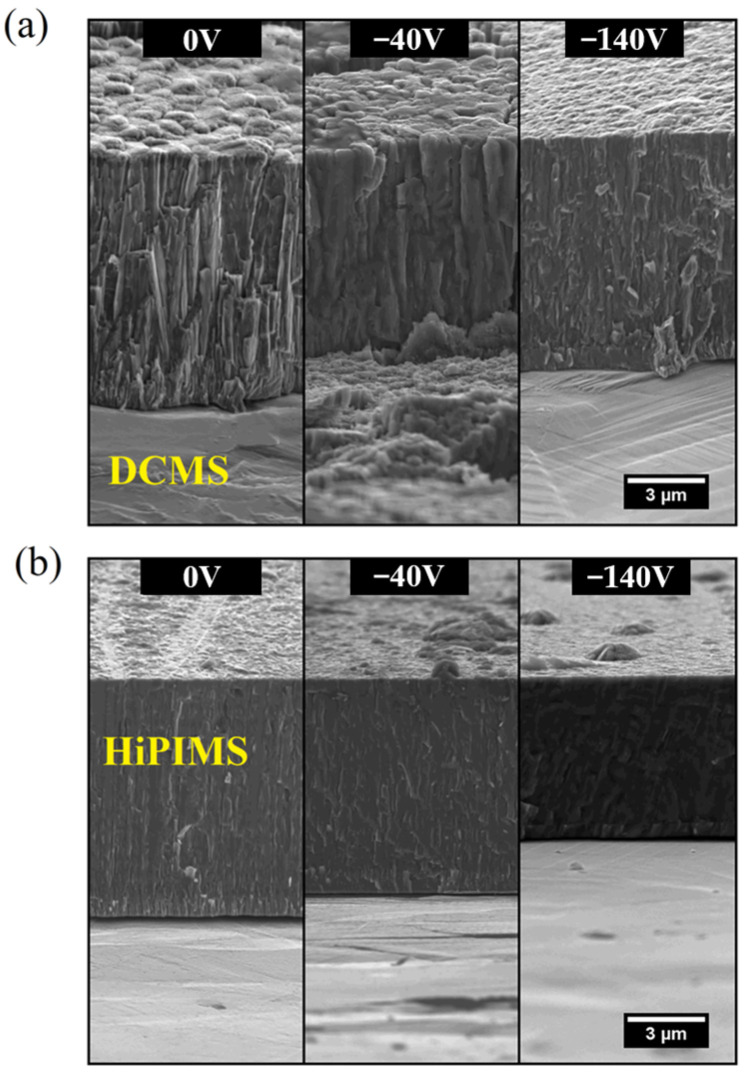
SEM micrographs of the cross-sectional morphologies of the CrN coating prepared by (**a**) DCMS and (**b**) HiPIMS [93]. The morphologies of the coatings obtained through the two techniques show significant differences, especially when influenced by the bias voltage. These disparities primarily result from the higher ionization rate inherent to HiPIMS and the applied bias voltage. Reprinted with permission from [93] Elsevier 2018.

Guimaraes et al. investigated the microstructure of CrN coatings grown by DCMS and HiPIMS under different bias voltages without heating, as shown in Figure 7 [93]. Figure 7a reveals that DCMS exhibits a coarse columnar crystal growth that gradually transitions into the dense columnar crystal morphology (from 0 V to −140 V), whereas CrN coatings grown using HiPIMS portray a denser structure. With increasing bias voltages, the dense columnar crystals gradually transition into nanocrystals (Figure 7b) and a featureless coating is observed (−140 V). Another concurrent phenomenon is re-sputtering, which also contributes to a reduction in the deposition rate of the material. 

The growth of coatings using HiPIMS is characterized by high ionic fluxes in the substrate. The ionic fluxes increase with increasing bias voltages. High-energy flux species reach the substrate, leading to rapid nucleation, suppressing the growth of columnar crystals and achieving dense polycrystalline or even nanocrystalline structures [50,100,101,102,103,104,105,106,107,108]. As discussed in Section 2.1, during the growth process, high-valence state plasma also acquires kinetic energy from the bias voltage, disrupting the continuous growth of columnar crystals and even re-sputtering loose grains, ensuring coating densification. Therefore, high-flux ionization excited by HiPIMS can surmount the typical low-density and rough microstructure, resulting in a unique morphology attained by low-temperature sputter deposition [33,40,109,110,111,112,113,114,115]. In comparison to coatings deposited by DCMS, HiPIMS imparted a higher hardness, lower friction coefficient, and better adhesion, wear resistance, and corrosion resistance to the coating [93]. 

## 4. The Influence of HiPIMS and DCMS Techniques on Mechanical and Corrosion Behaviors of CrN Coatings 

### 4.1. Hardness

The hardness of CrN coatings is determined by several factors. The composition and microstructure of the coating exert a significant influence. The nitrogen in the CrN coating enhances its hardness by forming a hard and wear-resistant nitride phase. Moreover, the crystalline structure of the coating, including the size and orientation of the grains, influences its hardness [116,117,118,119,120,121,122]. A dense and well-aligned structure typically results in higher hardness [121,122]. Conversely, the presence of defects within the coating could diminish its hardness. Therefore, controlling these factors during the deposition process is essential for attaining desirable hardness in CrN coatings. 

**Figure 8:**
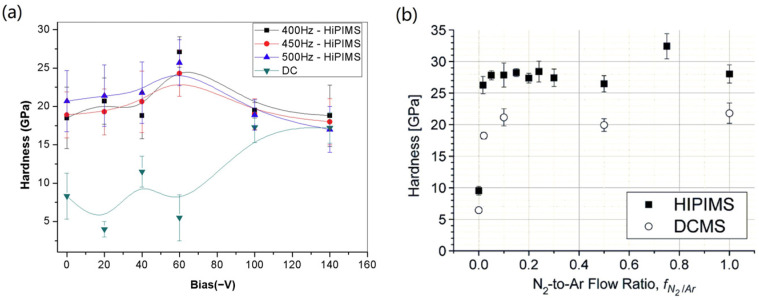
Under varying (**a**) bias voltage [93] and (**b**) f_N2/Ar_ conditions [49], the hardness of CrN coatings produced via HiPIMS surpasses that of DCMS. In the process of parameter adjustment, the hardness values of CrN coatings prepared by HiPIMS are consistently higher than those obtained through DCMS, which can be attributed to the higher ionization rate of the plasma stimulated by HiPIMS. Reprinted with permission from [93] Elsevier 2018, [49] IEEE Xplore 2010.

Guimaraes et al. [93] compared the performance of CrN thin coatings deposited using HiPIMS and DCMS (as in Figure 8a). They found that the hardness of CrN deposited by DCMS escalated with an increasing bias voltage. However, the hardness of CrN deposited by HiPIMS peaked at a bias voltage of 60 V and then decreased. This phenomenon might be due to the interactions of various parameters. The highly ionized plasma in HiPIMS, under a strong bias voltage, can inflict damage on the coating, thereby undermining its mechanical properties [114,123,124]. Nevertheless, the hardness of CrN deposited by DCMS consistently trailed that of CrN deposited by HiPIMS. Greczynski, G et al. compared the hardness values of CrN coatings deposited using HiPIMS and DCMS at N_2_-to-the Ar flow ratio f_N2/Ar_ [49]. The mechanical properties of the coatings deposited by the two sputtering techniques are compared in Figure 8b. HiPIMS yielded superior properties, even for metallic coatings (f_N2/Ar_ = 0), where nearly 50% harder coatings were achieved. For both sputtering techniques, the incorporation of a modest amount of nitrogen prompted a dramatic surge in the coating hardness. This phenomenon is widely recognized; the bonding strength of CrN ceramic phase coatings significantly exceeds that of metal bonds. The incorporation of both covalent and ionic bonds in the CrN ceramic phase resulted in stronger atomic interactions, thereby enhancing the material’s strength. Furthermore, the introduction of nitrogen causes lattice distortion and restricts the movement of dislocations. This lattice distortion and the constraint of dislocation movements contributed to the solid solution strengthening effect, resulting in the remarkable mechanical properties and wear resistance of CrN ceramic phase coatings [125,126,127]. With the increase in the N_2_-to-Ar flow ratio f_N2/Ar_, the hardness values of the coatings deposited by HiPIMS surpassed those deposited by DCMS [49]. The analysis in the article suggests that this can be attributed to the denser structure of the CrN coatings deposited by HiPIMS [49].

The hardness of CrN coatings significantly affects their mechanical properties. CrN coatings with higher hardness generally exhibited improved mechanical performance. A high hardness indicates that the coating has robust resistance against plastic deformation, wear, and scratching. This property is especially important for applications where the coating is subjected to mechanical stresses or abrasive environments. 

### 4.2. Tribological Properties

The wear resistance of CrN coatings is determined by several key factors. Firstly, the hardness of the coating is a critical determinant. A dense and well-aligned microstructure with a fine grain size tends to enhance wear resistance, as it can effectively distribute and absorb the applied forces [99,128,129,130,131,132,133]. The friction coefficient of the coating also exerts a significant influence on its tribological properties. The friction coefficient determines the resistance to relative motion between the coating and the counter surface. The friction coefficient of coatings is influenced by various factors, including coating composition, microstructure, surface roughness, and environmental conditions [134,135,136,137,138]. By carefully selecting and optimizing these factors, it is possible to improve wear resistance and extend the operational lifespan.

**Figure 9:**
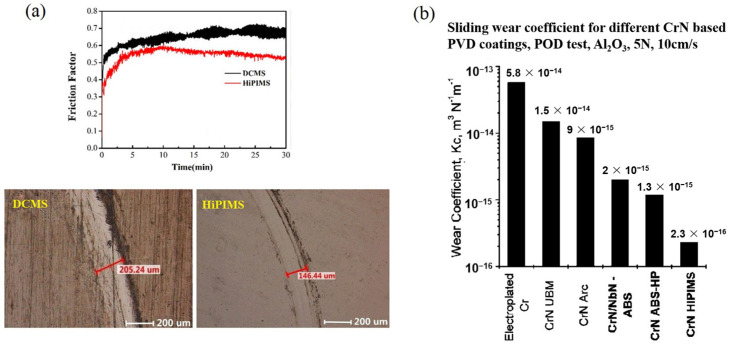
(**a**) The friction coefficient and wear groove width obtained from the friction and wear tests on CrN coatings prepared via HiPIMS and DCMS [48]. (**b**) The sliding wear coefficient of CrN coatings was prepared using various techniques [64]. Compared to DCMS, HIPIMS achieves a reduced friction coefficient and enhanced wear resistance. Reprinted with permission from [48] AIP Publishing 2020, [64] Elsevier 2003.

Li et al. conducted a comparative study on the friction coefficient and wear resistance of CrN coatings prepared with HiPIMS and DCMS [48]. The wear tracks were observed using an optical microscope, as shown in Figure 9a. CrN coatings deposited by HiPIMS exhibited lower friction coefficients compared to DCMS. This can be ascribed to the smoother surface and denser structure. By observing the wear tracks, they found that the wear scars of the CrN coatings deposited by HiPIMS were narrower. The analysis suggests that this is related to the improved crystallinity and higher hardness of the coatings prepared by HiPIMS. As shown in Figure 9b, Ehiasarian et al. reported the wear rates of Cr-based coatings deposited using different techniques [64]. CrN coatings obtained via HiPIMS exhibited wear rates two orders of magnitude lower than those prepared using DCMS. The decrease in the wear rate of CrN coatings can be attributed to several factors. Firstly, CrN coatings deposited by HiPIMS tend to feature a denser and more uniform microstructure, which enhances their resistance to wear. Secondly, the HiPIMS deposition process enables the formation of a smoother and more adherent coating surface, thereby reducing friction and minimizing wear. Furthermore, the higher hardness of CrN coatings obtained through HiPIMS contributes to their improved wear resistance. The combination of these factors, including improved microstructure, smoother surface, and higher hardness, collectively results in a significant reduction in the wear rate of CrN coatings deposited by HiPIMS compared to other deposition techniques, such as DCMS.

### 4.3. Electrochemical Behavior

The electrochemical corrosion performance of CrN coatings is influenced by a range of factors. In terms of the coating’s composition, CrN demonstrates exceptional corrosion resistance due to the formation of a protective chromium oxide (Cr_2_O_3_) layer on the surface, which acts as a barrier against corrosive environments and prevents interaction with the underlying material. Regarding the coating’s microstructure, factors like grain size, grain boundary condition, and density significantly impact the corrosion resistance of CrN coatings. Defects within the coating can reduce its inherent corrosion resistance, while various defects can also increase the likelihood of contact between the corrosive medium and the coating substrate, thereby impacting the overall corrosion resistance [137,138,139]. Hence, a fine-grained, well-structured, and compact microstructure generally enhances the coating’s corrosion resistance [19,21,139,140,141,142,143,144]. 

**Figure 10:**
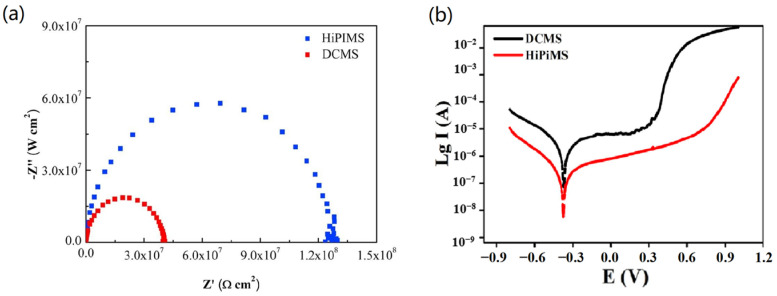
The (**a**) Nyquist plots [72] and (**b**) potentiodynamic polarization curves [48] of CrN coatings prepared via HiPIMS and DCMS were compared. The coating obtained through HiPIMS exhibits a larger capacitive arc radius and a lower corrosion current density, indicating better corrosion resistance. Reprinted with permission from [72] Elsevier 2020, [48] AIP Publishing 2020.

Zhang et al. [72] and Li et al. [48], respectively, deposited CrN coatings on ABS substrates and stainless steel substrates to compare the differences in electrochemical corrosion performance between HiPIMS and DCMS, as illustrated in Figure 10a,b. The capacitive arc radius of the CrN coating prepared by HiPIMS was significantly larger, and the polarization resistance (Rp) calculated using the fitting circuit model was 1.23 × 10^5^ KΩ·cm^−2^, which was higher than that of DCMS (3.95 × 10^4^ KΩ·cm^−2^). Table 1 presents the data obtained from Figure 10b, indicating that the corrosion current of the coatings deposited by HiPIMS was approximately one order of magnitude lower than that of the coatings deposited by DCMS. The analysis suggests that the microstructure of the CrN coatings prepared by DCMS was comparatively loose. The NaCl electrolyte infiltrated along the interfaces between grain columns, corroding the crystals and resulting in larger corrosion currents. On the other hand, CrN coatings prepared by HiPIMS exhibited a compact structure with fewer defects, making them more resistant to the corrosion of the crystals.

## 5. Prospects 

### 5.1. Innovative Use of HiPIMS Waveforms

**Figure 11:**
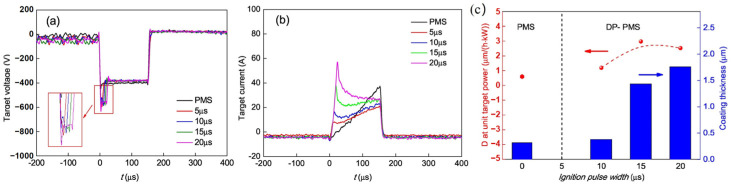
The (**a**) target voltage and (**b**) target current of the pulsed magnetron sputtering (PMS) and dual-pulse pulsed magnetron sputtering (DP-PMS) with different ignition pulse widths (inset in (**a**) shows the enlarged view). (**c**) The normalized static deposition rate (red data) and coating thickness (blue data) as a function of ignition pulse width for DP-PMS and PMS [145]. The ignition pulse can effectively improve the target discharge efficiency and deposition rate. Reprinted with permission from [145] Elsevier 2019.

**Figure 12:**
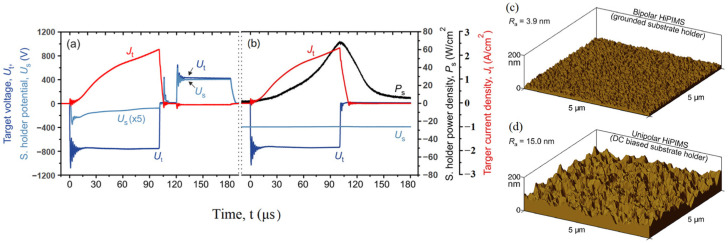
Waveforms of the target voltage, Ut (t), target current density, Jt (t), and substrate holder potential, Us (t), under (**a**) bipolar HiPIMS (positive pulse voltage of 400 V with the substrate holder at a floating potential) and (**b**) unipolar HiPIMS (with the substrate holder at −400 V) waveforms that additionally include the substrate holder power density, Ps(t). Surface morphology and roughness of CrN coatings prepared under the aforementioned conditions for both (**c**) bipolar HiPIMS and (**d**) unipolar HiPIMS [146]. CrN coatings prepared using bipolar HiPIMS exhibit smoother surfaces and lower roughness. Reprinted with permission from [146] IOPscience2020.

In recent years, numerous researchers have sought to optimize and enhance HiPIMS technology for the deposition of CrN coatings. For example, Wu et al. employed a pulse ignition technique in which an elevated voltage was introduced at the initiation of each pulse in HiPIMS to ignite the plasma [145], as depicted in Figure 11a. They explored the impact of ignition voltage pulse width on amplifying the ion flux of the plasma. The variation in target current (Figure 11b) suggests that the ignition pulse can effectively enhance the ion flux generated by HiPIMS, leading to an increased deposition rate of CrN coatings (Figure 11c). Addressing the challenge of plasma utilization, Š. Batková et al. incorporated a positive pulse following each pulse to drive the HiPIMS afterglow towards the substrate [146], as demonstrated in Figure 12a. As indicated in Figure 12, the introduction of the positive pulse heightened the plasma flux reaching the substrate, resulting in a denser CrN coating with a smoother surface.

Although these innovative approaches are still in their developmental stages, they offer invaluable insights for the industrial production of CrN coatings using HiPIMS technology.

### 5.2. Irradiation Resistance and Corrosion Protection for Liquid Heavy Metals

CrN coatings also exhibit excellent performances and wide applications in nuclear industries, demonstrating promising results on small Inconel 600 samples within the Halden reactor [147]. In addition to offering robust corrosion protection in supercritical water or other coolants, CrN coatings have the capacity to withstand the corrosion of liquid heavy metals, which makes them a candidate for use as a coolant in next-generation nuclear reactors [148]. Kurata et al. investigated the applicability of the CrN coating to an LBE (liquid Pb-Bi) environment at 450 °C and 550 °C [149]. They found that the CrN coating exhibited good compatibility in LBE during the corrosion test because of its compact structure and high thermal stability. This allows the CrN coating to effectively act as a barrier between the LBE and the substrate. The denser the CrN coating is, the better the protective effect against LBE [150]. As discussed in Section 4.3, it is anticipated that CrN coatings prepared by HiPIMS will exhibit a compact structure with fewer defects than DCMS, making them more resistant to the penetration of LBE.

**Figure 13:**
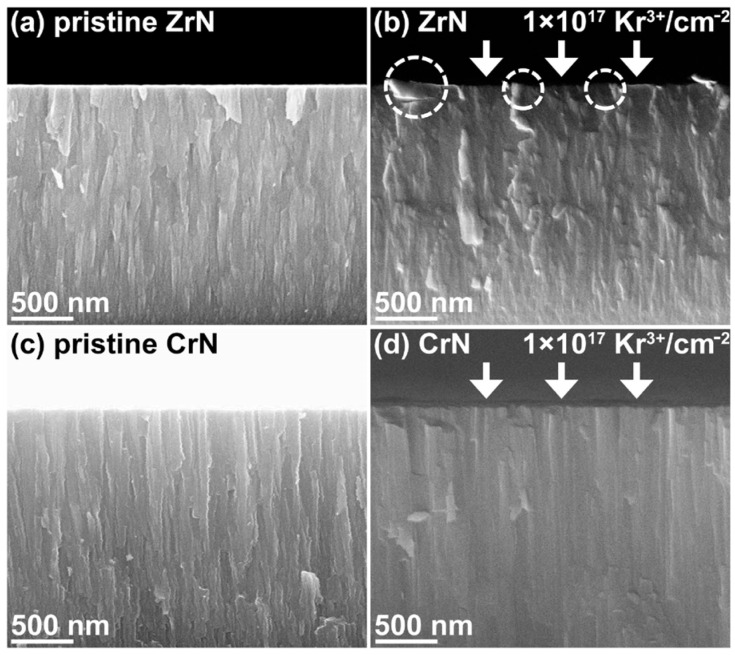
The cross-sectional SEM images of the ZrN and CrN coatings. (**a**,**c**) show the cross-sections of the ZrN and CrN, respectively. (**b**,**d**) illustrate the cross-sections of 600 keV Kr^3+^ ion-irradiated ZrN and CrN, respectively. The fluence of the ions was 1 × 10^17^ Kr^3+^/cm^2^ [157]. The microstructure of the CrN coating exhibited higher stability under Kr^3+^ ion irradiation when compared to ZrN. Reprinted with permission from [157] Elsevier 2019.

Nevertheless, the operational lifespan of structural materials applied in nuclear reactors can be diminished due to the bombardment from energetic particles. High doses of irradiation from energetic particles can inflict substantial damage to materials, leading to a precipitous rise in defect density and a marked deterioration in mechanical properties [151,152,153]. The accumulation of helium bubbles (a byproduct of transmutation) and voids resulting from vacancies can trigger significant swelling in irradiated materials, resulting in embrittlement and porosity [154,155,156]. As in Figure 13, Wu et al. deposited the ZrN and CrN coatings with DCMS and irradiated them with 600 keV Kr^3+^ at room temperature [157]. Their observations revealed an interruption in the continuity of columnar grains on the ZrN coating surface, attributable to the recrystallization and the grain coarsening of the ZrN. In contrast, the cross-sectional analysis of the irradiated CrN coating still manifested a persistent columnar structure. This evidence underscores CrN’s superior structural stability against irradiation when compared to ZrN. The CrN coating’s steadfast structure under irradiation conditions presents it as a viable candidate as a structural material in reactors. Furthermore, materials endowed with smaller grain sizes, and therefore higher grain boundary densities, are known to exhibit superior irradiation resistance. This resistance is primarily due to the interaction between irradiation-induced defects and defect sinks. Such interactions can immobilize or annihilate defects through various mechanisms, including diffusion, trapping, recombination, or annihilation reactions [158,159,160,161]. This preferential pathway for defect migration and removal fortifies the overall structural integrity of the material, augmenting its mechanical properties and resistance to degradation [151,152,153,154,155,156,157,158,159,160,161,162,163,164]. As elucidated in Section 3, the creation of such microstructural characteristics aligns perfectly with the capabilities of the HiPIMS technique. With denser structures and higher adhesion, it is anticipated that CrN coatings fabricated by HiPIMS will exhibit enhanced mechanical properties and irradiation resistance compared to those deposited by DCMS. 

## 6. Conclusions

In this review of recent literature, CrN deposition using both the HiPIMS and DCMS techniques was compared, and disparities in discharge, coating growth, and consequential mechanical properties were examined. Contrary to DCMS, HiPIMS induced a higher level of ionization in the Cr and N plasma. This enhanced ion flux characteristic fostered better crystallinity during the growth phase of CrN coatings. Through the efficient utilization of this high ion flux, it can become feasible to refine the grain size of the coating, yielding a dense or even nanocrystalline microstructure. Nevertheless, CrN coatings deposited by HiPIMS experience limitations such as reduced deposition rates and elevated internal stress. The internal stress can be judiciously managed by modulating the bias voltage, while recent advancements have sought to improve the low deposition rate by altering the HiPIMS pulse waveform. In the context of ceramic coatings like CrN, which are renowned for their superior properties, the formation of covalent bonds between Cr particles and N ions necessitates overcoming a substantial energy barrier. The highly ionized plasma in HiPIMS supplies the requisite energy for this process. The swift nucleation of high-flux coating-forming particles during coating growth sets the stage for attaining a dense microstructure. This microstructure accounts for the superior mechanical properties of CrN coatings grown by HiPIMS relative to those grown by DCMS. Table 2 summarizes the typical characteristics of CrN coatings prepared by two techniques. 

Attributable to their exceptional performance attributes, CrN coatings have garnered extensive applications across diverse sectors, including in medicine, electronics, and nuclear industries, in addition to their traditional usage in cutting tools. The specific demands of these fields, such as the intricate surface growth of coatings and the development of metal–ceramic multilayer structures, impose stricter stipulations on the properties of CrN coatings. There is an anticipation that HiPIMS technology, paralleled with the wide-ranging deployment of CrN coatings, holds significant promise to serve an expanded spectrum of industries and explore innovative possibilities in emerging application scenarios.

## Figures and Tables

**Table 1:** The results of the electrochemical experiments are based on Figure 10b.

	Samples	Ecorr (V)	Icorr (A/cm^2^)
The results of polarization curve fitting by Li et al. [48]	CrN (DCMS)	−0.37	2.8 × 10^−7^
	CrN (HiPIMS)	−0.34	2.75 × 10^−8^

**Table 2:** CrN coatings deposition techniques, morphology, and mechanical properties from previous studies.

		DCMS	HiPIMS
Deposition parameters [15,48,49,72,90,93]	Voltage (V)	200–500	600–1200
	Peak current (A)	0.5–10	80–600
	Peak power (kW)	0.5–4	8–400
Microstructure and morphology	Texture (substrates: Si (100)) [72]	Predominately (111)	Predominately (200)
	Crystal size (nm) [15]	28	8
	Residual stresses (MPa) [93]	−400	−1200
	Microstructure [72]	The nanocrystalline and amorphous composite structure	The compact nanocrystalline structure
	Morphology [48]	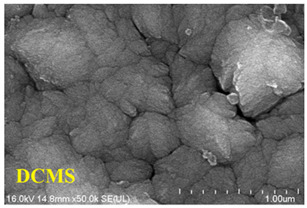 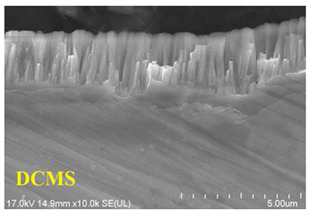	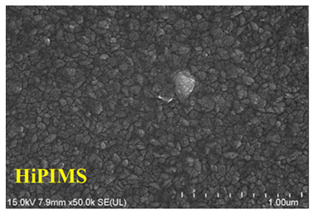 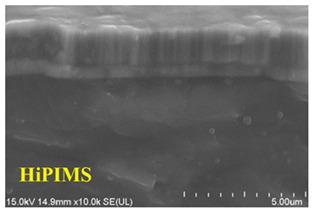
Mechanical property	Hardness (GPa) [72]	15	22
	Elastic modulus (GPa) [72]	240	270
	Friction coefficient [48]	0.64	0.545

## Data Availability

Not applicable.
